# Alpine vegetation dataset from three contrasting mountain ranges differing in climate and evolutionary history

**DOI:** 10.1016/j.dib.2019.104816

**Published:** 2019-11-15

**Authors:** Jesús López-Angulo, David S. Pescador, Ana M. Sánchez, Arantzazu L. Luzuriaga, Lohengrin A. Cavieres, Adrián Escudero

**Affiliations:** a Departamento de Biología, Geología, Física y Química Inorgánica, Escuela Superior de Ciencias Experimentales y Tecnológicas, Universidad Rey Juan Carlos, Móstoles, Spain; b Departamento de Botánica, Facultad de Ciencias Naturales y Oceanográficas, Universidad de Concepción, Concepción, Chile; c Instituto de Ecología y Biodiversidad (IEB), Santiago, Chile

**Keywords:** Alpine grassland, Cover, Dataset, Mediterranean and temperate mountains, Vegetation survey, Plant functional trait

## Abstract

Vegetation above treeline constitutes one of the most vulnerable ecosystems to climate warming and other drivers of Global Change. Given the panorama of such an uncertain future facing these plant communities, it is critical to know how they respond to environmental changes and improve the knowledge on the potential impacts of climate change on their distribution. Recently, with the impressive development of trait-based approaches, relevant progress has been made to better understand the relationships between environmental conditions and plant communities. In this data paper, we describe data on abundances of 327 alpine plant species across 430 subplots of 2.4 m × 2.4 m in three mountain ranges (Sierra de Guadarrama and Pyrenees in Spain, and the Central Andes in Chile). We provide data on different environmental variables that represent variation in abiotic conditions and operate at different spatial scales (e.g., climatic, topographic and soil conditions). Data on six plant functional traits are also shown, which were measured on ten individuals of each species, following standard protocols. We provided the dataset as tables in the supplementary material. This information could be used to analyse the relationship between the alpine vegetation and changes in environmental conditions, and ultimately, to understand ecosystem functioning and guide conservation strategies of theses threatened and valuable ecosystems.

**Table:** Specifications Table

Subject	Ecology
Specific subject area	Plant Community Ecology, Biodiversity, Plant Science
Type of data	Three datasets on vegetation, environmental conditions and functional trait data per plant species
How data were acquired	Field Survey
Data format	Raw data
Parameters for data collection	Field observation in summer during the flowering peak.
Description of data collection	Cover data from 327 species visually estimated; environmental data from 430 subplots coming from three different mountain ranges; and information from six plant functional traits per plant species
Data source location	Guadarrama National Park, Sierra de Guadarrama, central Spain. Ordesa - Monte Perdido National Park, Pyrenees, north Spain. Central Andes, Chile
Data accessibility	With the article in the supplementary material
Related research article	J. López-Angulo, D.S. Pescador, A.M. Sánchez, A.L. Luzuriaga, L.A. Cavieres, A. Escudero, Impacts of climate, soil and biotic interactions on the interplay of the different facets of alpine plant diversity, Sci. Total Environ. 698 (2020) 133960. https://doi.org/10.1016/j.scitotenv.2019.133960 [1]

**Table:** 

**Value of the Data** • The dataset can be used to carry out ecological studies analysing the relationship between alpine vegetation and changes in environmental conditions through taxonomic and functional information. • Due to the nested sampling design, with information across large gradients and structured across two scales (plot and subplot), the dataset is suitable to evaluate assembly processes at different spatial scales. • Given spatial coordinates are provided, changes in the vegetation can be assessed through time. • The dataset can be useful in conservation purposes and climate change studies, and it can be integrated into macroecological analyses and species distribution modelling. • Plots of 20 m side are commonly used in the literature, so this dataset can be useful for comparative studies of alpine plant diversity and assembly patterns. • Dataset provides information from remote areas with few if any previous recordings (especially Chile)

## 1 Data

The data presented in this paper involves: (1) abundance data from 327 species; (2) environmental data from 430 subplots coming from three different mountain ranges; and (3) data from six plant functional traits.

1. Vegetation data: Percentage cover of 327 plant species found in the 430 subplots (2.4 × 2.4 m) sampled across three mountain ranges differing in climate and evolutionary history (the Sierra de Guadarrama and the Pyrenees in Spain, and the Central Andes in Chile). We sampled vegetation above the treeline, in snow-free zones such as windblown slopes and fellfields, avoiding rocks, screes, snow beds or humid depressions. The vegetation was consistently patchy and dominated by grasses, creeping chamaephytes, perennial forbs and cushion-like plants. The vegetation data are available in the ‘AbundanceDATA’ sheet from the supplementary material.
2. Environmental data: We sampled vegetation in a wide range of aspects and slopes, and covering the complete elevation gradient in each mountain region. Elevation ranged from 1890 to 2420 m in Guadarrama, from 1650 to 2550 m in the Pyrenees, and from 2064 to 3627 m a.s.l. in the central Chilean Andes. We recorded the sampling dates, spatial coordinates, aspect and slope in each location sampled. We collected one soil sample in bare ground and another one in the vegetated area in each sampling unit. From soil samples, we estimated phosphatase (Phos) and β-glucosidase (Glu) soil enzymatic activities, soil organic carbon (SOC), total nitrogen (NT), available phosphorus (PT) and potassium (K) as key nutrients related to primary productivity and the build-up of nutrient pools [2]. We also measured soil pH and electrical conductivity. The environmental data are available in the ‘FieldDATA’ sheet from the supplementary material.
3. Trait data: We measured six functional traits associated with critical and well-known functional axes of plant performance for most of the species in each mountain range: vegetative height (VH), plant size (PS), specific leaf area (SLA), leaf dry matter content (LDMC), leaf thickness (LT) and seed mass (SM, except for Chile). The trait data are available in the ‘TraitDATA’ sheet from the supplementary material.

## 2 Experimental design, materials, and methods

**Fig. 1:**
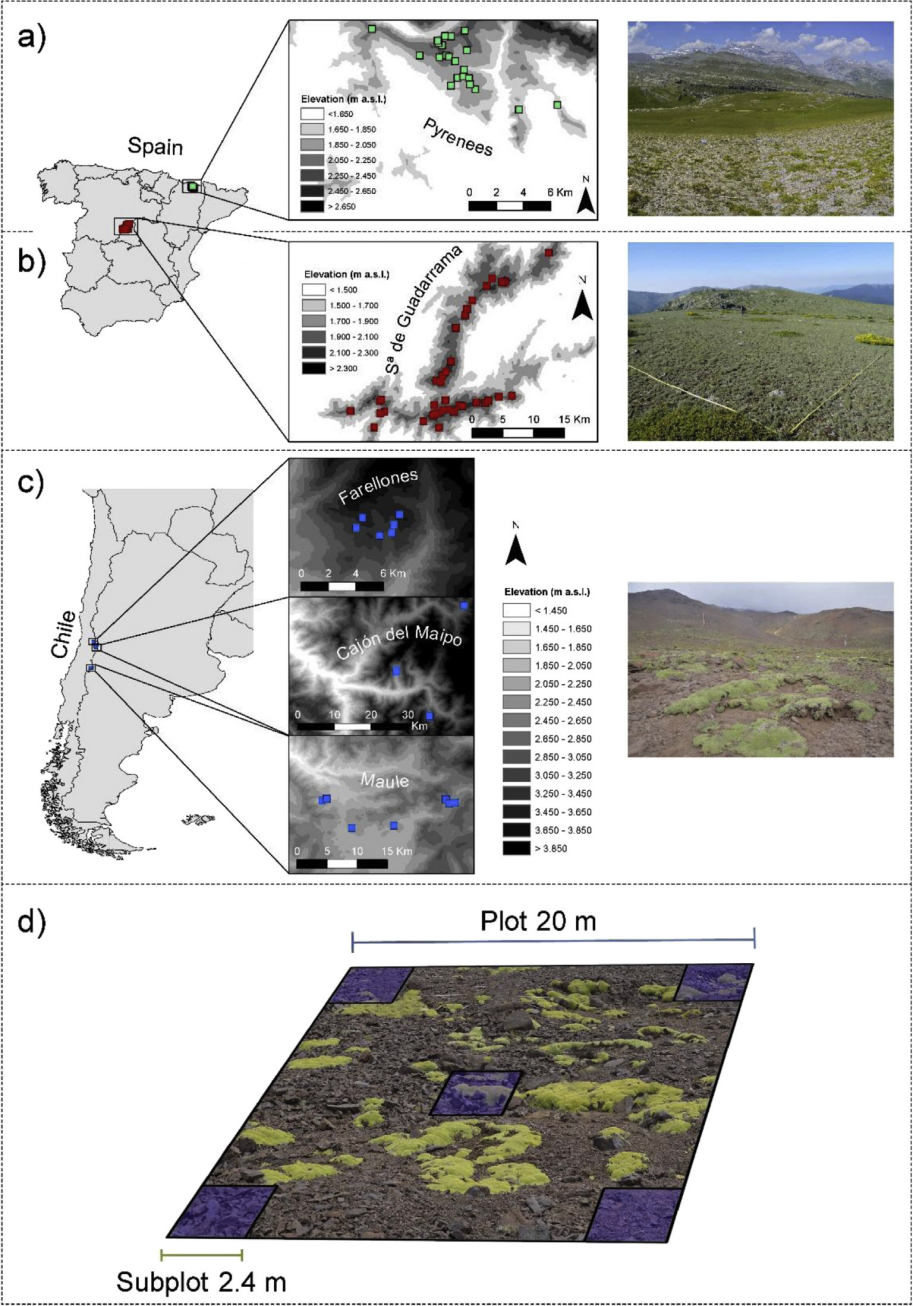
Sampling design. Locations of the three study areas across three mountain ranges differing in climatic conditions and evolutionary history: (a) Sierra de Guadarrama and (b) Pyrenees, in Spain; and (c) three different zones from the Central Andes in Chile. (d) Nested Sampling design structured across two scales (20 × 20 m Plot and 2.4 × 2.4 m Subplot).

We established 39 plots of 20 × 20 m (Fig. 1d) during June and July of 2011 in Guadarrama NP (Figs. 1a), 27 plots during July of 2013 and 2014 in Ordesa-Monte Perdido NP (Fig. 1b), and 20 plots during January of 2014 in three different areas [3] from the central Chilean Andes (Fig. 1c). We located four 2.4 m × 2.4 m subplots in the corners of each plot, and a fifth in the centre (Fig. 1d). This nested-plot design allowed comparisons of diversity across sites at different spatial scales. We visually estimated the percentage cover of each species at each 2.4 × 2.4 m subplot. We collected all the field data during the flowering peak. We obtained coordinates, elevation and aspect using a GPS (Garmin Colorado- 300; Garmin, Olathe, CO, USA) and slope using a clinometer (Silva Clinomaster; Silva Sweden, Sollentuna, Sweden). We used latitude, aspect and slope data to calculate Gandullo's potential solar radiation coefficient [4]:

$$PSR_{n}=sen\;i\cdot \cos\;p\;-\cos\;\alpha \cdot \cos\;i\cdot sen\;p$$

$$PSR_{s}=sen\;i\cdot \cos\;p\;+\cos\;\alpha \cdot \cos\;i\cdot sen\;p$$

where *PSR*_*n*_ is the potential solar radiation calculated in north-facing sites and PSR_s_ in south-facing sites, *i* is the solar incidence angle (i.e. 90° - latitude), *p* is the slope, *α* is the angle formed by the aspect and 0° for PSR_n_ and the aspect and 180° for *PSR*_*s*_.

We estimated the eight soil physicochemical properties, phosphatase and β-glucosidase enzymatic activities, soil organic carbon (SOC), total nitrogen, available phosphorus, potassium, pH and electrical conductivity for each soil sample. Then, we averaged and weighted the values of soil properties by the respective cover of bare ground and vegetated area as proxies of soil nutrient stocks in the soil. We determined soil organic C was by colorimetry after oxidation with a mixture of potassium dichromate and sulphuric acid. Total N and available P was determined on a SKALAR++ San Analyzer (Skalar, Breda, The Netherlands) in the Nutrilab/URJC lab after digestion with sulphuric acid and Kjedahl's catalyst [5]. We measured potassium (K) with the same analyser after the soil samples had been shaken with distilled water (1:5 ratio) for 1 h. We estimated β-glucosidase and acid phosphatase activities using the methodology described by Eivazi & Tabatabai [6] and Tabatabai and Bremner [7], respectively. We measured soil pH using a pH meter (GLP 21; Crison, Barcelona, Spain) and electrical conductivity with a conductivity meter (GLP 31; Cri-son, Barcelona, Spain) in a 1:2.5 mass:volume soil and water suspension. A detailed description of the estimation of eight soil properties is provided in Ref. [8].

We measured six plant functional traits in most of the plant species in the respective plant communities. These species represented at least 87% of the accumulated cover (99% in Guadarrama NP, 94% in Ordesa-Monte Perdido NP and 87% in central Chilean Andes). We sampled 10 randomly selected mature and healthy individuals of each species during the plot sampling according to standardized protocols [9]. We measured VH as the distance from the ground to the top of photosynthetic tissues and PS was calculated as PS=π·L·S/4 where *L* was the longest diameter and *S* was the shorter diameter perpendicular to the former one. We weighed two fresh well-developed leaves per individual using a microbalance (Mettler Toledo MX5, Columbus, OH; weight uncertainty ±1 μg). We estimated the projected area (≈leaf area) from each fresh leaf using a digital scanner (Epson Perfection 4870) and Adobe Photoshop CS4 software (Adobe Systems, San Jose, CA). We then oven-dried the leaves at 60 °C for 72 hours and measured dry mass using the microbalance. SLA was the ratio of the projected area divided by its dry mass and LDMC was the oven-dried mass of a leaf divided by its fresh water-saturated leaf mass. We estimated LT for each leaf as the mean of three measurements using a dial thickness gauge (Mitutoyo Co., Aurora, IL, USA). We measured seed mass in at least 30 dry seeds using the microbalance.
